# Pattern of Acute Poisoning and Drug Overdose Cases in the Emergency Department of a Tertiary Care Hospital: An Observational Study

**DOI:** 10.31729/jnma.v63i292.9257

**Published:** 2025-12-31

**Authors:** Lujaw Ratna Tuladhar, Suraj Sharma, Sanish Pokhrel, Nitin Dhamala, Uttam Lamichhane, Sushovit Karki

**Affiliations:** 1 Department of Pharmacology, Nepal Medical College and Teaching Hospital, Kathmandu, Nepal; 2 Department of Forensic Medicine, Nepal Medical College and Teaching Hospital, Kathmandu, Nepal; 3 Emmergency Department, Nepal Medical College and Teaching Hospital, Kathmandu, Nepal

**Keywords:** *acute poisoning*, *drug overdose*, *insecticide poisoning*, *intentional self-harm*, *wild honey*

## Abstract

**Introduction:**

Acute poisoning and drug overdose cases are common to emergency department visits worldwide, with variation according to regional factors. In Nepal, easy availability to agricultural chemicals and locally sold toxins such as wild honey present specific toxicological challenges. This study aims to observe the patterns of poisoning and drug overdose cases in a tertiary care hospital, including the distribution of accidental and intentional self-harm cases, types of poisoning agents, demographic characteristics, and clinical outcomes.

**Methods:**

A descriptive cross-sectional study was conducted from March 1 to November 30, 2024, at Nepal Medical College and Teaching Hospital after receiving ethical clearance (Ref. No. 41-080/081). All poisoning cases presented with acute poisoning or drug overdose during that time period were included. Data on age, gender, type of poison, and outcomes were compiled and analyzed using SPSS version 16. Descriptive statistics was applied.

**Results:**

Among 215 cases, 192 (89.30)% were accidental and 23 (10.70%) were intentional self-harm. Females accounted for 102 (53.10%) of accidental poisonings and 16(69.60%) of intentional self-harm cases. A total of 104 (48.40%) patients were in the age group of 19-39 years. Insecticides were the most common agents in both accidental 65 (33.90%) and intentional 12(52.20%) cases. Wild honey poisoning made up 23 (12%) of accidental exposures, especially in older adults. Hospitalization was required in 98 (51%) of accidental and 14 (60.90%) of intentional cases. Around 89 (41.39%) of patients left against medical advice. Mortality was low 2 (1%), narrowed to accidental cases only.

**Conclusions:**

The predominance of insecticide poisoning, female vulnerability, and the concentration of cases among young adults is alarming.

## INTRODUCTION

Acute poisoning and drug overdose cases are common problem worldwide, contributing significant cases to emergency department admissions.^1^ Poisoning cases varies geographically, influenced by local agricultural practices, cultural factors, and socioeconomic conditions. In Nepal, agricultural chemicals are easily available and locally traded region-specific toxic substances such as wild honey containing grayanotoxins pose unique challenges for toxicological management.^1^

Understanding the patterns of acute poisoning and drug overdose is important for effective prevention, timely clinical intervention, and mental awareness. The rationale of this study is to contribute data for our hospital to plan health strategies for regulating hazardous substances to reduce morbidity, mortality, and healthcare costs while improving hospital preparedness.

This study aims to observe the patterns of poisoning and drug overdose cases in a tertiary care hospital, including the distribution of accidental and intentional self-harm cases, types of poisoning agents, demographic characteristics, and clinical outcomes.

## METHODS

A descriptive cross-sectional study was conducted in the Emergency Department (ED) of Nepal Medical College and Teaching Hospital from 1st March to 30th November 2024 (9 months), following approval by the Institutional Review Committee (reference no. 41-080/081).

All 215 cases of acute poisoning and drug overdose cases presented to the Emergency Department during the 9-month study period were included using census sampling.

All cases of poisoning presented within 24 hours of ingestion or drug overdose during the study period were included. Poisoning agents and drugs were identified based on clinical history and verbal reports. Inclusion criteria included patients with confirmed acute poisoning or drug overdose who presented within 24 hours of ingestion and gave voluntary informed consent (or whose legal guardians gave consent) during admission in emergency department. Cases involving other medical conditions or refusal to give consent were excluded.

Data on demographics (age, gender), intent (accidental (unintentional, unexpectedly) or intentional self-harm), name of ingested poison or drug , and clinical outcomes were collected through medical records files in the emergency department. Accidental and intentional self-harm cases were identified through patient’s or guardian’s statement. Clinical outcomes recorded included hospital admission, discharge, referral, and discharge on patient request (DOPR), leave against medical advice (LAMA), and death.

Main variables analysed were age, gender, type of poisoning agent or drug, intentional or unintentional, and clinical outcome. Data were entered into SPSS version 16 and analysed descriptively using frequencies, percentages, and graphical presentations.

## RESULTS

Among 215 poisoning cases presented to the Emergency Department, there were 192 (89.30%) cases of accidental poisoning and 23 (10.70%) cases of intentional self-harm. There were 102 (53.10%) females with history of accidental poisoning and 16 (69.60%) females with history of intentional self-harm (Table 1).

**Table 1:** Gender based distribution of patients with accidental and intentional self-harm poisoning (n=215).

Gender	Accidental poisoning n(%)	Intentional-self-harm n(%)
Male	90(46.90%)	7(30.40%)
Female	102(53.10%)	16(69.60%)

**Table 2:** Age-group distribution of accidental and intentional self-harm poisoning cases (n=215).

Age group	Accidental poisoning n(%)	Intentional-self-harm n(%)
Infant (<1 year)	2(1.00%)	0(0%)
Toddler (1-4 years)	8(4.20%)	0(0%)
School age (5-12 years)	7(3.60%)	0(0%)
Adolescent (13-18 years)	16(8.30%)	4(17.40%)
Young adult (19-39 years)	92(47.90%)	12(52.20%)
Middle age (40-64 years)	55(28.60%)	7(30.40%)
Older adult (65+ years)	12(6.20%)	0(0%)

**Table 3:** Table showing pattern of poisoning cases among accidental and intentional self harm cases (n=215).

Poison	Accidental poisoning n(%)	Intentional-self-harm n(%)
Unknown	28(14.60%)	2(8.70%)
Insecticide	65(33.90%)	12(52.20%)
Rodenticide	20(10.40%)	1(4.30%)
Non-steroidal-anti-inflammatory drug	11(5.70%)	3(13.00%)
Multi-drug poisoning	11(5.70%)	2(8.70%)
Corrosive acid	9(4.70%)	1(4.30%)
Mushroom	11(5.70%)	0(0%)
CNS depressant	5(2.60%)	0(0%)
Wild honey	23(12%)	0(0%)
Carbon monoxide poisoning	3(1.60%)	0(0%)
Anti-depressant	1(0.50%)	1(4.30%)
Camphor	0(0%)	1(4.30%)
Kerosene poisoning	3(1.60%)	0(0%)
Food poisoning	2(1.0%)	0(0%)

**Table 4:** Table showing clinical outcome in poisoning cases (n=215).

Remarks	Accidental n(%)	Intentional-self-harm n (%)
Admitted	98(51.00%)	14(60.90%)
Leave against medical advice (LAMA)	80(41.70%)	9(39.10%)
Discharged	4(2.10%)	0(0%)
Referred	5(2.60%)	0(0%)
Discharged on patient request (DOPR)	3(1.60%)	0(0%)
Expired	2(1.0%)	0(0%)

Among 192 cases of accidental poisonings, there were 92 (47.90%) cases which belonged to 19-39 years age group, 55 (28.60%) cases which belonged to 40-64 years age group while there were 2 (1.00%) cases among infants (< 1 year). Among 23 cases of intentional self-harm, there were 12 (52.20%) cases which belonged to 19-39 years age group, 7 (30.40%) cases which belonged to 40-64 years age group. There were 0 (0%) cases of intentional self-harm among infants (<1 year) and toddlers (1-4 years) (Table 2).

Among 192 cases of accidental poisonings, there were 65 (33.90%) cases with insecticide use, 20 (10.40%) cases of rodenticide use, 23 (12.00%) cases of wild honey poisoning and 11 (5.70%) cases of Non-steroidal-anti-inflammatory drug overdose. Similarly, there were 28 (14.60%) cases of unknown drug use among accidental poisoning. Among 23 cases of intentional self-harm, there were 12 (52.20%) cases of insecticide use, 3 (13.00%) cases of non-steroidal-anti-inflammatory drug use and 2 (8.70%) cases of multidrug poisoning. Similarly, there were 2 (8.70%) cases of unknown drug poisoning among intentional self-harm (Table 3).

There were 112 (52.09%) admitted cases and 89 (41.39%) leave against medical advice (LAMA) cases among acute poisoning and drug overdose cases presented to the Emergency Department. Among accidental poisoning, there were 98 (51.0%) admitted cases, 80 (41.70%) LAMA cases and 3 (1.60%) discharged on patient request (DOPR) cases. Among intentional self-harm, there were 14 (60.90%) admitted cases and 9 (39.10%) LAMA cases (Table 4).

## DISCUSSION

This study provides important information’s on acute poisoning and drug overdose cases in Nepal’s tertiary care emergency setting. The rising number of cases compared to past data reflects a need for serious public health concern. Both accidental and intentional self-harm poisoning cases were reported more in females, possible reason could be dissatisfaction, not meeting their expectation, social pressure, economic burden, work overload and hectic lifestyle. Similarly, both accidental and intentional self-harm poisoning cases were reported in high number in young adults (19-39 years of age group). The pattern of poisoning showed that insecticides were the causative agent for accidental and intentional self-harm poisoning. Although most of the patient were admitted in the emergency department, leaving against medical advice (LAMA) were also in higher figure.

In a study conducted by Shrestha et al.^2^ (2011) reported 354 cases of poisoning in six years of duration at Nepal Medical College, whereas our study(2024), we observed 215 cases within just nine months. This data add to the growing body of evidences. A BMC study from 2021 reported a 13-fold increase in fatal pesticide self-poisoning between 1980 and 2019 in Nepal, attributing this trend to family conflicts, poor mental health services, and the widespread availability of toxic substances (insecticides) and over the counter medication.^3^ These are just the major cause behind the substantial rise in number of poisoning cases, the minor cause still needs to be addressed.^3^

Females represented a slightly higher proportion (53.40%) of poisoning and overdose cases, consistent with findings from a study conducted at Kathmandu medical college and teaching hospital in the year 2003 (female to male ratio was 1.09:1) and similar low and middle-income countries.^4,5^ For instance, Acharya et al.^6^ in their study conducted in western region of Nepal at Gandaki medical teaching hospital in the year 2015-2017 reported 71% of poisoning cases in females and in another study conducted by Shakya et al.^7^ at Kathmandu medical college in the year 2019-2020 reported 62.80% of the poisoning cases were in females. This gender disparity may be driven by psychosocial stressors, including marital conflict, gender inequality, and limited access to mental health support. Public health initiatives and support must be gender-sensitive, focusing on early identification of women at risk and provide mental health services, reinforcement, skills and laws for domestic violence.^8^

Young adults (19-39 years) constituted the majority (48.37%) of poisoning cases. This is consistent with several other Nepalese studies that identify this age group as particularly vulnerable. A study conducted in Kathmandu medical college and teaching hospital in the year 2003 reported 38.80% of the poisoning cases in the age group 21-30 years.^4^ Another study conducted at Patan Hospital in the year 2023 reported 47.61% of the poisoning cases were seen in the age group 18-29 years.^9^ Another study conducted at KIST medical college in the year 2021 reported 39.06% of the poisoning cases belonging to the age group of 21-30 years.^10^ Similarly a study conducted at Tribhuvan university teaching hospital and Bharatpur district hospital in the year 2002 reported higher incidence of poisoning in the age group 16-25 years and below 15 years respectively.^11^ Contributing factors likely include lack of opportunities after higher education, low income with high expenditure, difficulty in continuing family occupation, occupational stress, family disputes, substance misuse, slow growth in career and underlying mental health issues.^8^ The involvement of young adults is particularly concerning, as this demographic is critical for long-term societal and economic development. Prevention strategies focusing on this age group may help reduce poisoning rates.

Insecticide poisoning was the most common, particularly involving organophosphates, cypermethrin, and other agricultural chemicals. Intentional ingestion accounted for 52.20% of insecticide cases, highlighting the continued use of these agents in suicidal attempts. In a study conducted by Lohani et al.^12^ in the year 2002 reported zinc phosphide as a common poison that was reported in Nepal drug and poison information center; and in another study conducted at Bir hospital by Singh D^13^ in the year 2005 reported insecticide (63%) as the common cause of poisoning, emphasizing the role of easy access and poor regulation.

Just like insecticides, rodenticides particularly zinc phosphide and bromadiolone, which were responsible for 10.30% of poisoning cases. These agents are easily accessible household products, often used intentionally for self-harm. Their continued use underscores the need for regulated sales and safe storage practices.^11^ Rodenticides are available in every local shop and marts because of high demand. Due to infestation of mice and rats in every house, people purchase them to protect their crops and prevent damage to household equipment’s. However consumers of such rodenticides are not aware of its proper use and instructions to keep such rodenticides away from the hands of children’s.

A unique finding in our study was the significant proportion (12%) of wild honey poisoning. The presence of grayanotoxins in wild honey derived from Rhododendron species represents a region specific toxicological challenge. Public education campaigns are needed to inform communities about the dangers of consuming unprocessed or wild honey.^14,15^ Honey is available and sold on the streets and in shops under different brand names, each claiming to be organic, chemical-free, and extracted from forests, thereby creating an attractive appeal for consumers. This type of wild honey is believed to have higher medicinal and nutritional value.

In a study conducted at Kathmandu medical college and teaching hospital in the year 2003 and 2008, children (13.40%, 9.40%) were particularly susceptible to accidental poisoning, most commonly from kerosene.^4^ Similar trends have been documented in studies from KIST and other centers, with accidental ingestion accounting for over 90% of paediatric cases . Enhancing caregiver education and promoting child safe packaging of household chemicals are critical and minimal preventive measures that can be taken.^16^ In spite of kerosene having pungent smell and unpleasant taste, kerosene poisoning is still common poisoning in children. Even a small swallow of kerosene can cause aspiration into the lungs (chemical pneumonitis), which is far more dangerous than the systemic toxicity. Children may spit it out immediately because of the bad taste, but by then aspiration may have already occurred.

Pharmaceutical agents involved in overdoses included CNS depressants, NSAIDs, antidepressants and multi-drug poisoning. Although these cases were fewer in number, they are clinically significant due to their potential for delayed toxicity and severe complications. Acharya et al.^6^ reported a strong association between psychiatric illness and drug overdose, particularly anxiety and depression. Greater availability at home increases the chances of accidental or intentional ingestion because they already have access to antidepressants, overdose is a frequent method of attempted suicide. Children may ingest antidepressants unintentionally if medicines are not stored properly.

Mushroom (2.70%) and carbon monoxide poisoning (0.70%) were rare but notable. These agents are often seasonal or environment-specific.^17^ Public health efforts should address foraging safety and indoor pollution risks, respectively.^18^ Corrosive substance ingestion (4.10%) and poisoning from unidentified substances (15.10%) present additional challenges in diagnosis and management, necessitating improved toxicology support in emergency settings.^18^ In most of the ingestions, household cleaning products were used. Enforcing regulations for the manufacturers of household cleaning products, labelling proper way of handling the chemical (use of rubber gloves, keep out of fire instructions) can significantly reduce the incidence of this potentially fatal condition.^19^

Nearly half of the patients (52.09%) required hospitalization, reflecting the severity of cases. But 41.39% of patients left the facility against medical advice (LAMA), a figure higher than expected. Another study reported, 37.5% were admitted to ICU , 12.50% referred elsewhere, and 50% left against medical advice. This highlights challenges in patient retention and the need for better counselling and support.^20^ Contributing factors may include financial constraints, stigma, psychological distress, or distrust in medical institutions. Future qualitative research is needed to explore the cause in details. Referrals (2.7%) and deaths (1.4%) emphasize the need for improved emergency preparedness and prompt interventions. Strengthening poisoning management protocols and expanding critical care capacity could help reduce mortality and morbidity. Financial support for poisoning cases from ward offices, metropolitan can prevent the number of LAMA cases.

This study is subject to several limitations, including its singlecentre design, possible underreporting, lack of toxicological confirmation in all cases, and limited follow-up. Additionally, psychosocial assessments were not systematically performed, which may have led to under-recognition of mental health comorbidities.

## CONCLUSION

This study highlights the growing burden and shifting patterns of acute poisoning and drug overdose. The predominance of insecticide poisoning, female vulnerability, and the concentration of cases among young adults is alarming. Addressing the high rate of LAMA requires investigation of socioeconomic and psychological factors affecting patient adherence.

## Data Availability

The data are available from the corresponding author upon reasonable request.
